# Cancer cases and deaths attributable to lifestyle risk factors in Chile

**DOI:** 10.1186/s12885-020-07187-4

**Published:** 2020-07-25

**Authors:** Leandro F. M. Rezende, Eliana Murata, Beatriz Giannichi, Luciana Yuki Tomita, Gabriela Arantes Wagner, Zila M. Sanchez, Carlos Celis-Morales, Gerson Ferrari

**Affiliations:** 1 Universidade Federal de São Paulo, Escola Paulista de Medicina, Departamento de Medicina Preventive, Sao Paulo, SP Brazil; 2 Centro de Investigación en Fisiología del Ejercicio - CIFE, Universidad Mayor, Santiago, Chile; 3 Institute of Cardiovascular and Medical Sciences, University of Glasgow, Glasgow, UK; 4 Laboratorio de Rendimiento Humano, Grupo de Estudio en Educación, Actividad Física y Salud (GEEAFyS), Universidad Católica del Maule, Talca, Chile; 5 Laboratorio de Ciencias de la Actividad Física, el Deporte y la Salud, Facultad de Ciencias Médicas, Universidad de Santiago de Chile – USACH, Estación Central, 7500618 Santiago, Chile

**Keywords:** Epidemiology, Cancer, Lifestyle, Chile

## Abstract

**Background:**

To identify modifiable risk factors that contribute to cancer holds important public health relevance for setting up prevention strategies. Therefore, the aim of this study was to estimate the proportion of cancer cases and deaths attributable to alcohol consumption, high body mass index (BMI), low fruits and vegetables consumption, lack of physical activity, tobacco smoking, and passive smoking in Chile in 2018.

**Methods:**

We retrieved data from a national representative survey to describe the distribution of six lifestyle risk factors. Relative risks of each risk factor-cancer pair were obtained from published meta-analysis and pooled cohort studies. Cancer cases and deaths were obtained from the GLOBOCAN 2018.

**Results:**

Nearly 30% of all cancer cases (15,097 out of 50,320 cases) and 36% of all cancer deaths (10,155 out of 28,010 deaths) in Chile in 2018 were attributable to lifestyle risk factors. Smoking and high BMI accounted for most of the cancer cases (9232 and 4394, respectively) and deaths (6868 and 2572). The cancer burden of other lifestyle risk factors varied by sex. In men, the proportion of all cancer cases attributed to alcohol were 3.7% compare to 2.0% for women. Cancers cases and deaths of the larynx, lung, oral/cavity, esophagus and bladder could be at least halved if lifestyle risk factors were eliminated.

**Conclusion:**

Smoking and high BMI were the leading causes of preventable cancer cases and deaths within the six lifestyles factors considered. Cancer prevention strategies should consider evidence-based interventions and public policies to encourage the adoption of a healthier lifestyle.

## Background

Although cardiovascular diseases have been the primary cause of death in Chile over the last two decades, nowadays cancer is the leading cause of death and disability-adjusted life years [1, 2]. In 2018, 53,365 cancer cases were diagnosed and 28,443 cancer deaths occurred in Chile [3]. Most common incident cancers were prostate, stomach, and lung for men, breast, cervix uteri, and gallbladder for women [1]. Compared to other high-income countries, the age-standardized incidence rates for combined cancers are lower in Chile (304.7 vs 195.5 per 100.000 persons-year), whereas cancer mortality rates are similar (96.8 vs 95.7 per 100.000 persons-year) [3]. On the other hand, both cancer incidence and mortality are higher in Chile than in other Latin American and Caribbean countries (189.6 per 100.000 for cancer incidence; 86.5 per 100.000 for cancer mortality) [3]. These differences in cancer rates between populations could be partially attributable to rapid changes in lifestyle risk factors that have occurred during the last decades in Chile [4–6].

Epidemiological and molecular studies have shown that cancer is caused by an interplay of several environmental, lifestyle and biological factors [7]. However, current evidence supports that around 30 to 50% of all cancers are attributable to modifiable risk factors [8–13]. Over the last decade, cancer burden attributable to modifiable risk factors (aka, the population attributable fraction [PAF]) has been estimated in several countries for setting priorities for cancer prevention strategies [8, 9, 11–15].

Currently, 74% of the Chilean population is overweight or obese, 33% are smokers, 12% engage in harmful alcohol use, 24% are physically inactive, and 85% eat less than 5 portions of fruit and vegetables a day [16]. Together, these lifestyle risk factors have been associated with higher risk of at least 19 cancer sites [17–19]. Despite regular national surveys on the prevalence of risk factors, evidence on cancer cases and deaths attributable to lifestyle risk factors in Chile is lacking. Such information could inform future public health policies and interventions aiming to reduce cancer occurrence.

The aim of this study was to estimate the proportion and number of cancer cases and deaths for 19 cancer sites attributable to lifestyle risk factors using nationally representative data on exposures and cancer occurrence in Chile in 2018.

## Methods

### Study design

We designed a study using national Chilean data from multiple sources. We retrieved the distribution of six lifestyle risk factors (alcohol consumption, high body mass index - BMI, low fruits and vegetables consumption, lack of physical activity, tobacco smoking and passive smoking) by sex using data from a national representative survey in Chile (Table 1) [6]. Relative risks (RR) of each risk factor-cancer pair by sex were retrieved from published meta-analysis and pooled data analysis of cohort studies [9, 14, 18, 20–35]. Estimated number of cancer cases and deaths (excluding nonmelanoma skin cancer) in adults 20 years or older in Chile in 2018 by sex and cancer site were retrieved from the GLOBOCAN 2018 [3]. Similar methodological approach has been used to estimate the burden of cancer attributable to modifiable risk factors in other countries [8, 9, 11–15].

**Table 1:** Distribution (proportion or mean and 95% confidence intervals) of lifestyle risk factors associated with cancer incidence and mortality in Chile

Exposure and distribution and theoretical minimum risk exposure level (in italic)	Men	Women	Cancer sites-related (ICD-10)
**Alcohol consumption (%)**			
*Abstainer (0 g/day)*	20.5 (18.7–22.5)	44.1 (42.2–45.7)	Lip, oral cavity, pharynx (C00-C14); Esophagus (C15; squamous cell carcinoma only); Colorectum (C18-C20); Liver (C22); Gallbladder (C23); Pancreas (C25); Larynx (C32); female Breast (C50)
Light (1–12.5 g/day)	31.5 (29.3–33.6)	41.4 (39.8–43.2)	
Moderate (12.6–49.9 g/day)	39.1 (37.0–41.5)	13.8 (12.6–14.8)	
Heavy (≥50 g/day)	8.8 (7.6–10.2)	0.7 (0.4–1.0)	
**High body mass index (in kg/m**^**2**^**)**			
Mean and standard deviation (*22 kg/m*^*2*^*and 1 sd*)	28.42 (28.20–28.65)	29.62 (29.43–29.83)	Esophagus (C15; adenocarcinoma only); Stomach (C16.0; cardia only); Colorectum (C18-C20); Liver (C22); Gallbladder (C23); Pancreas (C25); female Breast (C50; postmenopausal cancers only); Corpus uteri (C54-C55); Ovary (C56); Kidney, renal pelvis (C64-C66); Thyroid (C73); Multiple myeloma (C90). Prostate (C61; advanced only)
**Low fruits and vegetables consumption (%)**			
*≥400 g/day*	11.6 (10.3–12.9)	14.7 (13.6–15.9)	Oral cavity/pharynx (C00-C14); Larynx (C32)
300–399 g/day	9.1 (7.9–10.4)	11.9 (10.9–13.0)	
200–299 g/day	18.1 (16.4–19.8)	19.8 (18.5–21.1)	
100–199 g/day	38.0 (35.9–40.2)	36.9 (35.4–38.4)	
0–99 g/day	23.2 (21.5–25.9)	16.6 (15.4–17.8)	
**Low fruits consumption only (%)**			
*≥250 g/day*	6.2 (5.2–7.2)	6.5 (5.7–7.3)	Lung, bronchus. Trachea (C33-C34)
200–249 g/day	6.1 (5.1–7.2)	7.9 (7.1–8.8)	
150–199 g/day	11.2 (9.9–12.7)	15.4 (14.3–16.5)	
100–149 g/day	7.7 (6.5–8.9)	6.1 (5.4–6.9)	
50–99 g/day	27.5 (25.6–29.4)	30.5 (29.0–32.0)	
0–49 g/day	41.2 (39.1–43.5)	33.6 (32.1–35.1)	
**Lack of physical activity (%)**			
*≥8000 MET-min/week*	0.6 (0.3–0.9)	0.2 (0.1–0.4)	Colon (C18); female Breast (C50; post-menopausal cancers only)
4000–7999 MET-min/week	5.1 (4.1–6.1)	1.8 (1.4–2.2)	
600–3999 MET-min/week	23.6 (21.9–25.4)	13.7 (12.6–14.8)	
< 600 MET-min/week	70.7 (68.8–72.7)	84.3 (83.1–85.5)	
**Passive smoking (%)**			
*No*	82.7 (81.0–84.3)	87.0 (85.9–88.1)	Lung, bronchus, trachea (C33-C34)
yes	17.3 (15.7–19.0)	13.0 (11.9–14.1)	
**Smoking (%)**			
*Never*	37.7 (35.6–39.8)	52.6 (51.0–54.1)	Oral cavity/pharynx (C00-C14); Esophagus (C15); Stomach (C16); Colorectum (C18-C20); Liver (C22); Pancreas (C25); Nasal cavity/paranasal sinus (C30-C31); Larynx (C32); Lung, bronchus, trachea (C33-C34); Cervix (C53); Kidney, renal pelvis, ureter(C64-C66); Urinary bladder (C67); Myeloid leukemia (C92)
Former	28.9 (27.0–30.9)	21.6 (20.4–22.9)	
Current	33.4 (31–3-35.4)	25.8 (24.4–27.1)	

Importantly, we considered in our estimates only lifestyle risk factors with strong/convincing evidence for increasing the risk of cancer according to the International Agency for Research on Cancer (IARC) [17, 18] and the World Cancer Research Fund (WCRF) [19], and for which exposure data were available in Chile and dose-response relationship of exposures and site-specific cancers were well-defined (Table 1).

#### Assessment of lifestyle risk factors

We used data from the National Health Survey of Chile 2016–2017 (*Encuesta Nacional de Salud* - ENS), a national representative, population-based, household survey that enrolled 6233 participants over 15 years old [6]. ENS 2016–2017 sampling strategy considered a stratified, multistage and clustered random sample of households at the national, regional (15 Chilean geographical regions), urban and rural level. One participant per household was randomly selected. The sample size was calculated with a relative sampling error of less than 30% and an absolute sampling error of 2.6% to the national level. The data collection was performed between August 2016 and March 2017 [6]. In this study we included 5834 adults aged ≥20 years who responded to a self-reported questionnaire about alcohol consumption, weight and height, fruits and vegetables consumption, physical activity, tobacco smoking, and passive smoking. The ENS 2016–2017 was funded by the Chilean Ministry of Health and approved by the Ethics Research Committee of the School of Medicine at the Pontificia Universidad Católica de Chile (No. 16–019). Participants signed an informed consent to take part in the study. Details about ENS 2016–2017 are available elsewhere [6].

Alcohol consumption was assessed through average number of drinks in a regular day. One drink of beer, one glass of wine or one shot of distilled spirit was assumed to have 12.5 g of pure alcohol. Self-reported weight and height were obtained to calculate the BMI. Fruits and vegetables consumption were calculated based on the average frequency (days/week) and number of servings per day (i.e., each serving was defined as 80 g). Physical activity was assessed through the Global Physical Activity Questionnaire which include self-reported frequency (days/week) and duration (minutes) of active transport (walking and cycling), and moderate and vigorous recreational and occupational activities. We assigned the following metabolic equivalent tasks (MET) to each of these activities: 4 for active transport (walking and cycling), 3.8 for moderate and 7.8 for vigorous occupational activities; and 3 for moderate and 6 for vigorous recreational activities; and then calculated total physical activity (MET-minutes/week) [36]. Smoking was assessed based on current and prior tobacco use (never, former and current). Passive smoking among never smokers (yes, no) was defined based on regular exposure to smoke at home. Although lifestyle risk factors data were available by age-group, we decided to calculate the prevalence estimates by sex only in order to align with relative risk and estimated cancer occurrence data.

### Relative risks and estimated cancer cases and deaths

We obtained RR of each exposure-cancer pair by sex from published meta-analyses and pooled cohort studies of observational studies, which were used to estimate the burden of cancer attributable to lifestyle risk factors in other countries [9, 14, 18, 20–32, 34, 35]. The RR values were reported in a previous study [13]. Estimated number of cancer cases and deaths by sex and cancer sites (excluding nonmelanoma skin cancer) for adults aged ≥20 years from Chile in 2018 were retrieved from the GLOBOCAN 2018 [3]. Cancer occurrence were available by age group, but we obtained data by sex only to align information across data sources. Number of cases were estimated by modelling, using mortality: incidence ratios derived from five local cancer registries across Chile. Number of deaths between 2006 and 2015 was used to project cancer deaths in 2018. Details about cancer incidence and death in Chile are available elsewhere [37]. Whenever pertinent and available, we considered the association between lifestyle risk factors and cancer risk specific by subtype, stage (e.g., high BMI and advanced prostate cancer) (17) or period of life (e.g., lack of physical activity and postmenopausal breast cancer) (28). Proportion of cardia (32% in men; 22% in women) and non-cardia stomach cancers (68% in men; 78% in women), and esophagus adenocarcinoma (15% in men; 14% in women) and squamous cell carcinoma (85% in men; 86% in women) in Chile were obtained from previous studies [38, 39] and applied to GLOBOCAN estimates. We considered number of advanced prostate cancer cases equals prostate cancer deaths; breast cancer cases older than 50 years old as postmenopausal breast cancer.

### Data analysis

PAF by cancer site, sex, and exposures were calculated using the following equations:

PAF for categorical exposures:

$$ PAF=\frac{\sum_{i=1}^n{P}_i\ {RR}_i-{\sum}_{i=1}^n{P}_i^{\ast }\ {RR}_i\ }{\sum_{i=1}^n{P}_i{RR}_i} $$

where Pi is the proportion of the population at the level i of exposure, P*i represents a counterfactual scenario of theoretical minimum risk exposure level (i.e., 100% of the population in the lowest risk category of exposure), and RRi is the relative risk of cancer at the level i of exposure (Table 1).

PAF for continuous exposure (i.e., BMI):

$$ PAF=\frac{\int \mathrm{RR}\left(\mathrm{x}\right)\mathrm{P}\left(\mathrm{x}\right)\mathrm{dx}-\int \mathrm{RR}\left(\mathrm{x}\right){P}^{\ast}\left(\mathrm{x}\right)\mathrm{dx}}{\int \mathrm{RR}\left(\mathrm{x}\right)\mathrm{P}\left(\mathrm{x}\right)\mathrm{dx}} $$

where P is mean and standard deviation (sd) of BMI (in kg/m^2^), P* is the theoretical minimum risk exposure level (i.e., mean BMI of 22 kg/m^2^ and 1 sd), RR is the relative risk of cancer per 1 kg/m^2^ increase, and dx indicates the integration according to BMI units. Log-logit function was used to represent the dose-response relationship between BMI and cancer risk [38, 40].

Finally, to estimate the proportion of each cancer site attributable to combined lifestyle risk factors we used the combined PAF equation assuming that risk factors are independent (had no statistical interaction):

$$ Combined\  PAF=1-\prod \limits_{i=1}^n\left(1-{PAF}_i\right) $$

where PAF*i* is each lifestyle risk factor-cancer site PAF.

We summed the number of cases and deaths attributable to combined lifestyle risk factors across cancer sites. To obtain the overall proportion of attributable cancers, we divided the summed number of avoidable cancer cases and deaths by the total number of cancer cases and deaths, respectively.

## Results

### Cancer incidence

**Fig. 1:**
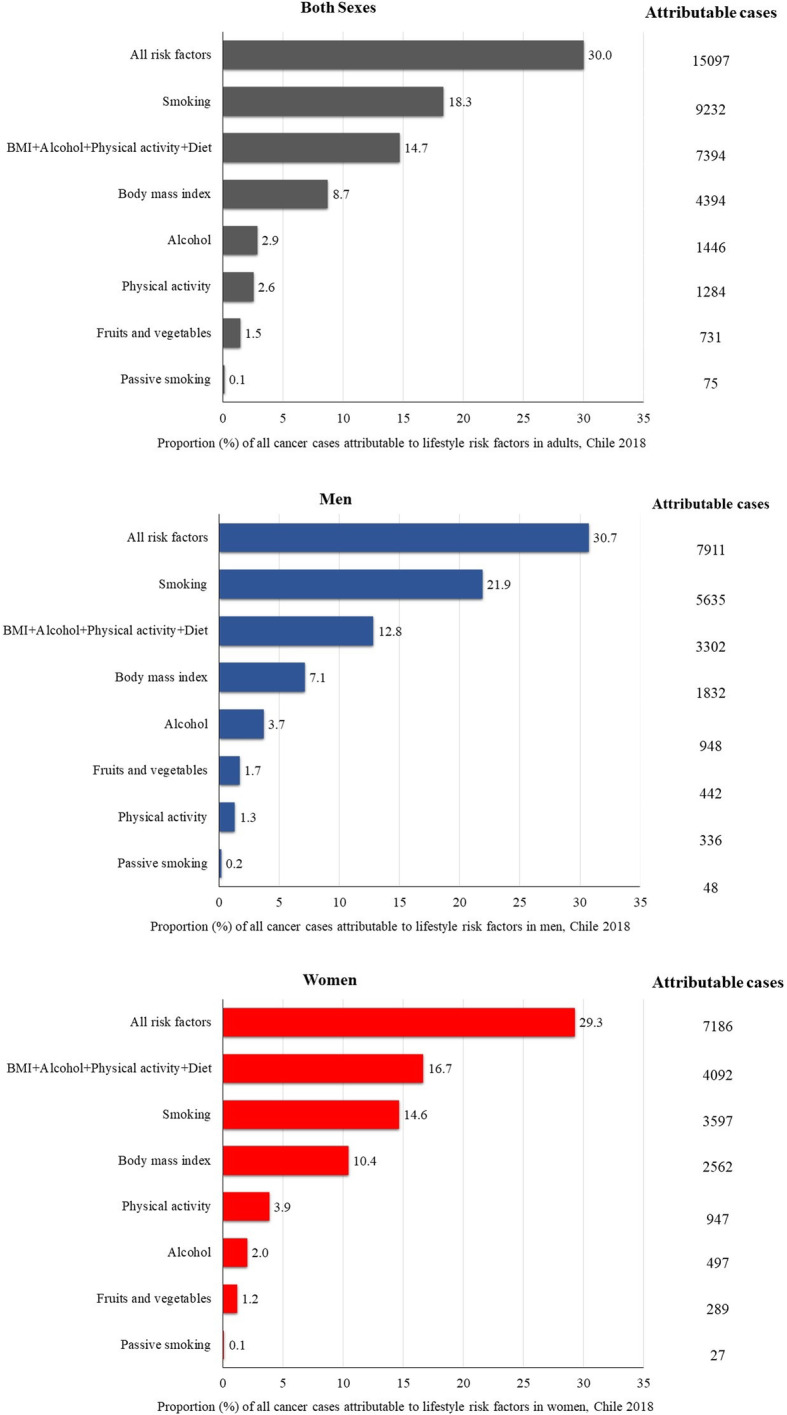
Proportion and number of all cancer cases attributable to lifestyle risk factors in Chile in 2018, by exposure and sex.

Nearly 30% of all cancer cases (15,097 out of 50,320 cases) that occurred in Chile in 2018 were attributable to lifestyle risk factors. The proportion was similar between men (30.7%) and women (29.3%) (Fig. 1).

Out of six lifestyle factors investigated, tobacco smoking was the most important factor, accounting for 18.3% of all cancer cases (*n* = 9232 cases) in Chile in 2018. The proportion was higher in men (21.9%) than in women (14.6%), reflecting higher prevalence of current smokers in the former group (33.4% vs 25.8%) (Fig. 1). Cancer sites with the highest PAFs for smoking were lung, larynx, and oral cavity/pharynx (Table 2).

**Table 2:** Proportion and number of cancer cases attributable to lifestyle risk factors in Chile in 2018 by exposure, sex, and cancer site

	Men			Women			Both		
Exposure/cancer site	Total number of cases	PAF (%)	Attributable cases	Total number of cases	PAF (%)	Attributable cases	Total number of cases	PAF (%)	Attributable cases
**Smoking**									
Lung	2163	90.7	1963	1708	87.3	1492	3871	89.2	3454
Larynx	225	85.7	193	28	82.2	23	253	85.3	216
Oral cavity/pharynx	299	63.9	191	219	59.1	129	518	61.9	321
Esophagus	412	58.9	243	372	56.8	211	784	57.9	454
Bladder	964	57.9	558	382	50.7	194	1346	55.8	752
Liver	866	36.7	317	709	18.6	132	1575	28.5	449
Stomach	3429	30.8	1056	1733	16.8	291	5162	26.1	1348
Cervix	–	–	–	1546	25.4	392	1546	25.4	392
Kidney	1215	29.2	354	705	8.7	61	1920	21.6	415
Pancreas	794	16.7	133	841	21.6	182	1635	19.2	314
Myeloid leukemia	522	29.4	153	514	4.5	23	1036	17.1	177
Colorectum	2952	16.1	474	2821	16.5	466	5773	16.3	940
**High body mass index**									
Corpus uteri	–	–	–	933	44.8	418	933	44.8	418
Kidney	1215	26.7	324	705	29.4	207	1920	27.7	531
Gallbladder	851	22.1	188	1848	26.8	496	2699	25.3	683
Liver	866	20.5	177	709	23.2	165	1575	21.7	342
Colorectum	2952	16.7	492	2821	10.8	304	5773	13.8	796
Pancreas	794	13.4	107	841	12.1	102	1635	12.7	208
Breast	–	–	–	5391	11.2	603	5391	11.2	603
Multiple myeloma	434	11.5	50	389	8.7	34	823	10.2	84
Thyroid	173	17.1	29	875	6.3	55	1048	8.1	85
Ovary	–	–	–	826	7.5	62	826	7.5	62
Stomach	3429	7.1	242	1733	5.5	95	5162	6.5	338
Esophagus	412	6.5	27	372	6.1	23	784	6.3	50
Prostate	6574	3.0	195				6574	3.0	195
**Alcohol consumption**									
Oral cavity/pharynx	299	44.6	133	219	11.1	24	518	30.5	158
Esophagus	412	39.7	163	372	16.8	63	784	28.8	226
Larynx	225	25.9	58	28	7.9	2	253	23.9	61
Gallbladder	851	15.3	130	1848	5.8	107	2699	8.8	237
Liver	866	8.4	73	709	5.1	36	1575	6.9	109
Colorectum	2952	12.1	356	2821	1.1	32	5773	6.7	388
Breast	–	–	–	5391	4.2	229	5391	4.2	229
Pancreas	794	4.2	34	841	0.7	6	1635	2.4	39
**Lack of physical activity**									
Colorectum	2952	11.4	336	2821	15.2	428	5773	13.2	764
Breast	–	–	–	5391	9.6	520	5391	9.6	520
**Low fruits and vegetables consumption**									
Larynx	225	25.2	57	28	23.2	7	253	25.0	63
Oral cavity/pharynx	299	25.2	75	219	23.2	51	518	24.4	126
**Low fruits consumption**									
Lung	2163	14.3	310	1708	13.6	232	3871	14.0	542
**Passive smoking**									
Lung	2163	2.2	48	1708	1.6	27	3871	1.9	75

*PAF* population attributable fraction

High BMI was responsible for 8.7% of all cancer cases (4394 out of 50,320 cases), with a higher proportion in women (10.4%) than in men (7.1%) (Fig. 1). Corpus uteri, kidney, and gallbladder cancers had the highest PAFs for BMI in women, and kidney, gallbladder and liver in men (Table 2).

Alcohol consumption and lack of physical activity were the third and fourth greatest preventable causes of cancer incidence, respectively, although the rank varied by sex. In men, the proportion of all cancer cases attributed to alcohol were 3.7% compare to 1.3% for lack of physical activity. In women, on the other hand, PAFs were 3.9% for lack of physical activity and 2.0% for alcohol consumption. The remaining factors, low fruits and vegetables consumption and passive smoking, contributed less than 2% each. The combined PAF for alcohol consumption, high BMI, lack of physical activity and low fruits and vegetables consumption was higher than attributable cases of smoking in women (16.7% vs 14.6%), but not in men (12.8% vs 21.9%) (Fig. 1).

**Fig. 2:**
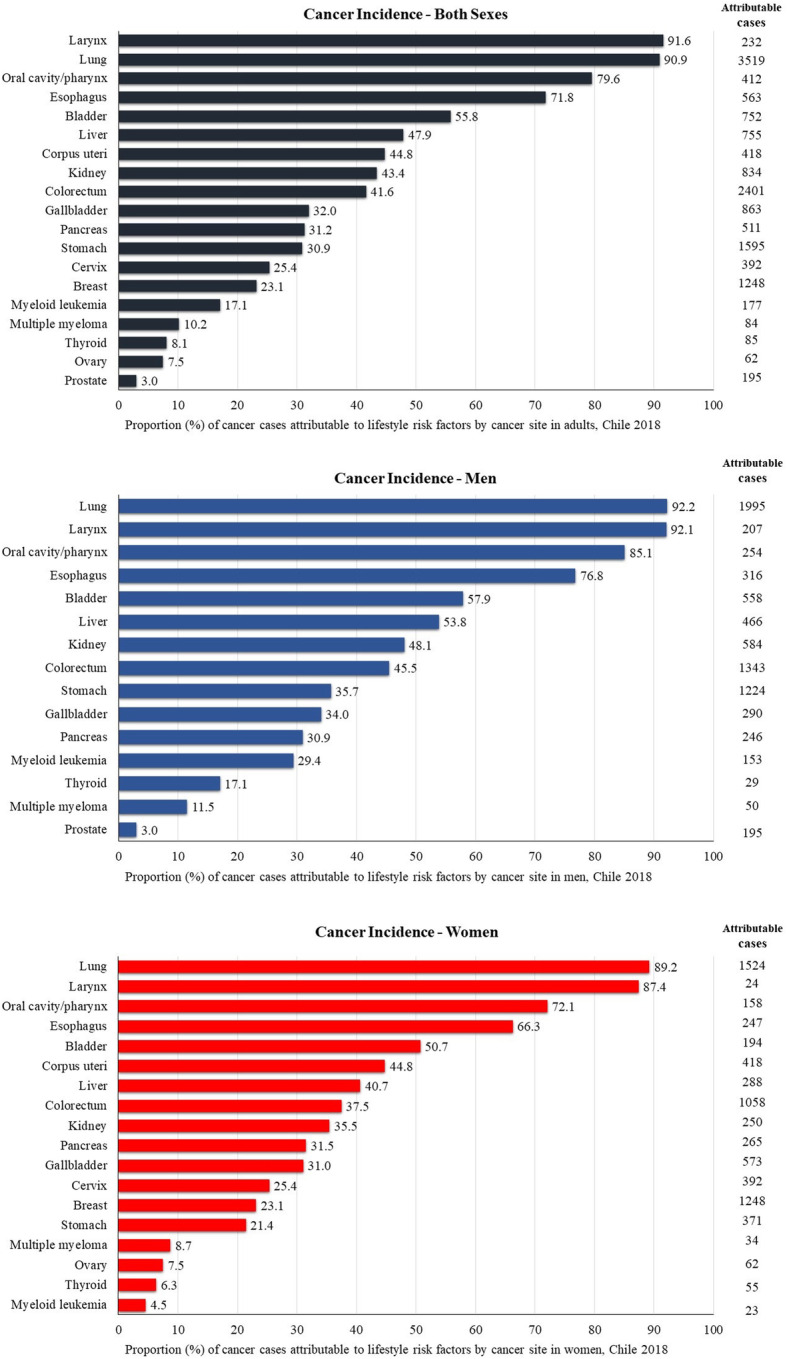
Proportion and number of cancer cases attributable to lifestyle risk factors in Chile in 2018, by cancer site and sex.

Proportion of site-specific cancer cases attributable to all six lifestyle risk factors ranged from 3.0% for prostate to 92.2% for lung in men, and 4.5% for myeloid leukemia to 89.2% for lung in women. Considering the absolute numbers (attributable cases), the most preventable cancer sites were lung cancer (1995 cases), colorectum (1343 cases), and stomach (1224 cases) in men, and lung (1524 cases), breast (1248 cases) and colorectum (1058 cases) in women. Incidence of larynx (91.6%), lung (90.9%), oral/cavity (79.6%), esophagus (71.8%) and bladder (55.8%) cancers could be reduced by half if these six lifestyle risk factors were eliminated (Fig. 2).

### Cancer mortality

**Fig. 3:**
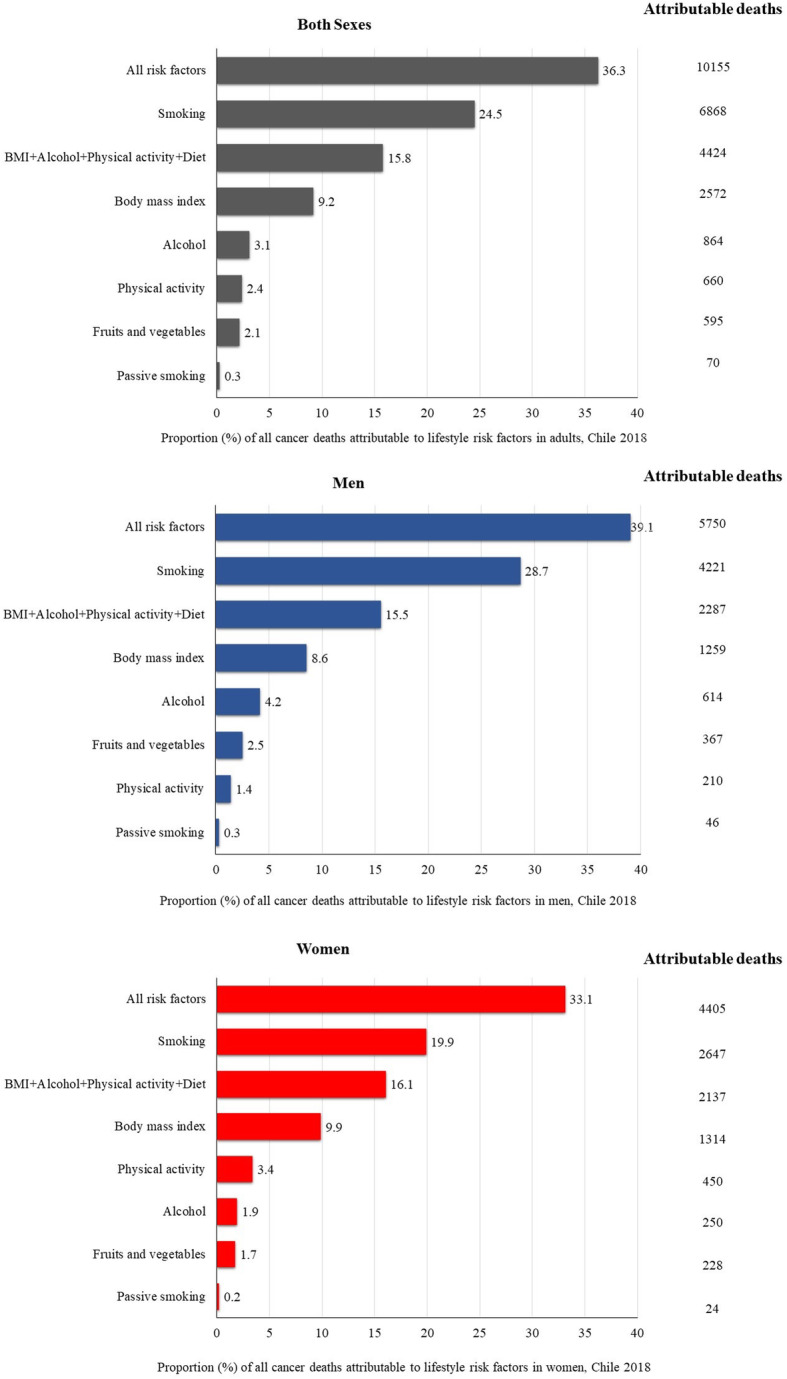
Proportion and number of total cancer deaths attributable to lifestyle risk factors in Chile in 2018, by exposure and sex

Lifestyle risk factors were responsible for 36.3% of all cancer deaths (10,155 out of 28,010 deaths) in Chile (Fig. 3). The proportion of cancer deaths was higher in men (39.1%) than women (33.1%) (Fig. 3).

The ranking of cancer mortality due to lifestyle risk factors was similar to cancer incidence. Smoking accounted for most of the cancer deaths (24.5%), followed by high BMI (9.2%), alcohol consumption (3.1%), lack of physical activity (2.4%), low fruits and vegetables consumption (2.1%) and passive smoking (0.3%). Similar to cancer incidence estimates, smoking and high BMI accounted for most of the cancer deaths in men (28.7 and 8.6%, respectively) and women (19.9 and 9.9%). The third highest estimated PAF was observed for alcohol consumption for men (4.2%) and lack of physical activity for women (3.4%) (Fig. 3).

**Fig. 4:**
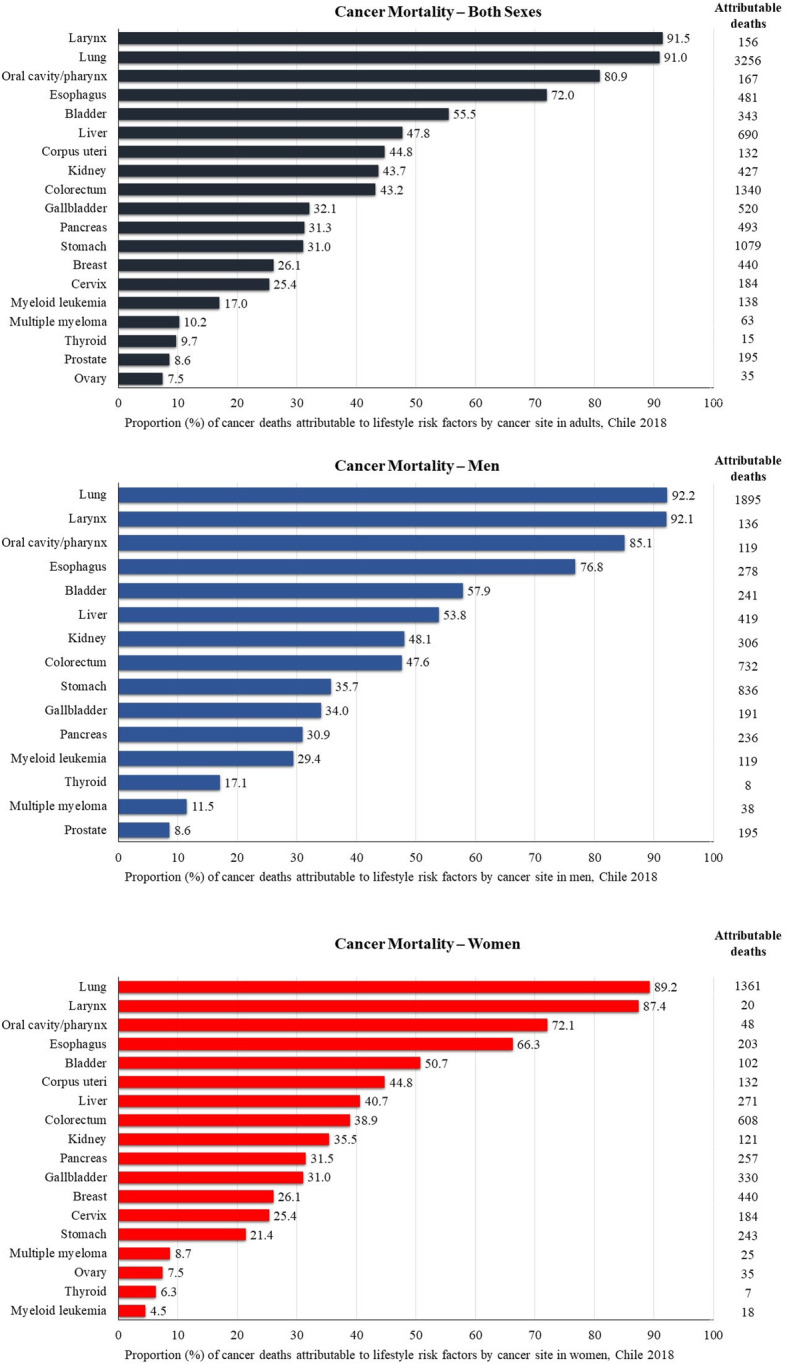
Proportion and number of cancer deaths attributable to lifestyle risk factors in Chile in 2018, by cancer site and sex

Cancer sites with the highest proportion of deaths attributed to lifestyle risk factors were larynx, lung, oral cavity/pharynx, esophagus, and bladder for both men and women. Cancer sites with highest absolute attributable deaths were lung (1895 deaths), stomach (836 deaths) and colorectum (732 deaths) for men, and lung (1361 deaths), colorectum (608 deaths), and breast (440 deaths) for women (Fig. 4). Proportion and number of cancer deaths by sex, exposures, and cancer sites are displayed in Table 3.

**Table 3:** Proportion and number of cancer deaths attributable to lifestyle risk factors in Chile in 2018, by exposure, sex, and cancer site

	Men			Women			Both		
Exposure/cancer site	Total number of deaths	PAF (%)	Attributable deaths	Total number of deaths	PAF (%)	Attributable deaths	Total number of deaths	PAF (%)	Attributable deaths
**Smoking**									
Lung	2055	90.7	1865	1525	87.3	1332	3580	89.3	3197
Larynx	148	85.7	127	23	82.2	19	171	85.2	146
Oral cavity/pharynx	140	63.9	89	66	59.1	39	206	62.4	129
Esophagus	362	58.9	213	306	56.8	174	668	57.9	387
Bladder	417	57.9	241	201	50.7	102	618	55.5	343
Liver	778	36.7	285	666	18.6	124	1444	28.3	409
Stomach	2342	30.8	721	1136	16.8	191	3478	26.2	912
Cervix	–	–	–	725	25.4	184	725	25.4	184
Kidney	636	29.2	185	342	8.7	30	978	22.0	215
Pancreas	763	16.7	127	816	21.6	176	1579	19.2	304
Myeloid leukemia	406	29.4	119	402	4.5	18	808	17.0	138
Colorectum	1538	16.1	247	1562	16.5	258	3100	16.3	505
**High body mass index**									
Corpus uteri	–	–	–	295	44.8	132	295	44.8	132
Kidney	636	26.7	170	342	29.4	100	978	27.6	270
Gallbladder	560	22.1	124	1063	26.8	285	1623	25.2	409
Liver	778	20.5	159	666	23.2	155	1444	21.7	314
Colorectum	1538	17.8	274	1562	11.2	175	3100	14.5	449
Breast	–	–	–	1688	13.0	220	1688	13.0	220
Pancreas	763	13.4	102	816	12.1	99	1579	12.7	201
Multiple myeloma	328	11.5	38	284	8.7	25	612	10.2	63
Thyroid	49	17.1	8	106	6.3	7	155	9.7	15
Prostate	2270	8.6	195				2270	8.6	195
Ovary	–	–	–	469	7.5	35	469	7.5	35
Stomach	2342	7.1	165	1136	5.5	63	3478	6.6	228
Esophagus	362	6.5	23	306	6.1	19	668	6.3	42
**Alcohol consumption**									
Oral cavity/pharynx	140	44.6	62	66	11.1	7	206	33.9	70
Esophagus	362	39.7	144	306	16.8	51	668	29.2	195
Larynx	148	25.9	38	23	7.9	2	171	23.5	40
Gallbladder	560	15.3	86	1063	5.8	61	1623	9.1	147
Liver	778	8.4	65	666	5.1	34	1444	6.9	99
Colorectum	1538	12.1	186	1562	1.1	18	3100	6.6	203
Breast	–	–	–	1688	4.2	72	1688	4.2	72
Pancreas	763	4.2	32	816	0.7	5	1579	2.4	38
**Lack of Physical activity**									
Colorectum	1538	13.6	210	1562	16.7	261	3100	15.2	470
Breast	–	–	–	1688	11.2	190	1688	11.2	190
**Low fruits and vegetables consumption**									
Larynx	148	25.2	37	23	23.2	5	171	25.0	43
Oral cavity/pharynx	140	25.2	35	66	23.2	15	206	24.6	51
**Low fruits consumption**									
Lung	2055	14.3	294	1525	13.6	207	3580	14.0	501
**Passive smoking**									
Lung	2055	2.2	46	1525	1.6	24	3580	2.0	70

*PAF* population attributable fraction

## Discussion

Nearly 30% of all cancer cases and 36% of all cancer deaths in Chile in 2018 were attributable to six lifestyle risk factors. Smoking and high BMI were the leading causes of preventable cancer cases and deaths within the six lifestyle risk factors studied. The cancer burden attributable to lifestyle risk factors varied by sex, reflecting differences in the exposure patterns between men and women and sex-specific associations (e.g., lack of physical activity and breast cancer in women). Five cancer sites could be reduced by half if lifestyle risk factors were eliminated.

To our knowledge, this is the first study to estimate the burden of cancer attributable to several lifestyle risk factors in Chile. Likewise other high-income countries and Latin American countries, smoking, high BMI and alcohol consumption were the major causes of preventable cancer in Chile [8–10, 12, 13]. Country-wide PAF for cancer incidence using similar methodological approach showed that smoking-related cancer were higher in Chile (18.3%) than in the Canada (17.5%), United Kingdom (UK) (15.1%), Australia (13.4%) and Brazil (15.5%), but not in the United States of America (USA) (19.0%) [8, 9, 12, 13, 15]. Chile also had the highest PAF for high BMI (8.7%), followed by USA (7.8%), UK (6.3%), Brazil (4.9%), Australia (3.4%), and Canada (3.1%) [8, 9, 12, 13, 15]. The proportion of cancer cases attributable to alcohol consumption was lower in Chile (2.9%) than in the USA (5.6%), Brazil (3.8%), and UK (3.3%), similar to Australia (2.8%), and higher than in Canada (1.8%) [8, 9, 12, 13, 15].

Our findings may be timely and useful for the recently published Chile’s National Cancer Prevention strategies (NCP) 2018–2028 [41]. The NCP 2018–2028 include several strategic lines of action, including “promotion, education and primary prevention”, where lifestyle risk factors are one of the main topics proposed to curb the burden of cancer. In this regard, public policies and interventions to reduce tobacco smoking, high BMI and alcohol are imperative. Since 2006, Chile has implemented several policies to control tobacco, which reduced the prevalence of smoking from 39.8% in 2009/2010 to 32.5% in 2016/2017 [16]. Strengthening these successful public policies, while accounting for new challenges to tobacco control (e.g., regulation of flavored and candy-like tobacco product) [42] is important to achieve the NCP 2018–2028 goals. However, the prevalence of tobacco smoking is still higher in Chile than in other Latin American countries [43]. Possible explanations for that are the lack of complete attendance and enforcement of World Health Organization’s (WHO) MPOWER strategy (i.e., stands for Monitor, Protect, Offer, Warn, Enforce, and Raise), especially with inappropriate low taxation in the country [44, 45].

Different from smoking, the prevalence of overweight (≥BMI 25 kg/m^2^) increased around 18% from 2003 (61%) to 2016/2017 (72.2%) [16, 41, 46]. In response to this obesity epidemic, several lines of actions on diet and physical activity have been proposed by the Chile Ministry of Health, such as increase in sugar-sweetened beverage tax to 18%, prohibition of unhealthy food sales and marketing in schools, and labeling of unhealthy foods containing high levels of calories, sugar, sodium or saturated fat [47]. A recent before-and-after study found that purchases of sugar-sweetened beverage significantly declined after the Chile’s Law of Food Labeling and Advertising [48]. Since 2002, the Chile’s National Policy on Physical Activity and Sports have aimed to amplify community-based physical activity programs and sports groups, disseminate the benefits of physical activity for health, and develop sports elite groups. Consequently, physical activity among adults Chileans have slightly increased from 26.4% in 2006 to 31.8% in 2015 [49]. Further reductions in the consumption of ultra-processed drinks and foods and promotion of physical activity are necessary for obesity control [50, 51] and, consequently, cancer prevention [52, 53].

Alcohol consumption among Chilean population dropped from 1960 to 1990. In 2018, the mean consumption of alcohol reached 9.3 L/year per capita, a value 16% superior to the America Region mean (8.0 L) [54]. The prevention of alcohol-attributable cancers depends on the population supporting policy efforts to reduce alcohol consumption. Thus, to accelerate the progress, WHO launched the SAFER framework, aiming to support governments in taking practical steps to implement evidence-based interventions to reduce the harmful use of alcohol [55]. Moreover, it is essential to promote public awareness about the risks between alcohol use and certain cancer sites since, unlike tobacco, alcohol is not dread as a possible cause of cancer by the general population [56].

Our study has several limitations. First, high quality, long-term prospective cohort studies on cancer etiology are inexistent in Chile, although ongoing cohort studies will certainly be useful in the near future (i.e., The Maule Cohort study – MAUCO). Therefore, we used RR from meta-analysis and pooled data of observational studies from other high-income countries. Whether these RR are applicable to Chilean population is unknown and warrants further investigation. The RR, prevalences of lifestyle risk factors and estimates of cancer cases and deaths were extracted by sex only, without considering other socio-demographic differences, such as age, ethnicity, and socioeconomic status. Second, we used the most recent nationally representative data on exposures profile in Chile (2016–2017), which may have not properly considered the latency between lifestyle risk factors and cancers. A similar methodological approach has been used in previous country-wide PAF estimates in the USA [9], China [14] and Brazil [13]. Although this approach accounts for the most recent exposure profile, depending on the trends in lifestyle risk factors and cancer occurrence over time, this may have biased our results. For instance, PAF for smoking may be underestimated because the prevalence of smoking has decline in recent years, while PAF for high BMI may be overestimated do to increase in the prevalence of overweight. Moreover, the estimated prevalence of lifestyle risk factors assumes that ENS coverage was equally distributed throughout the Chilean population. Moreover, some of the behaviors can be more influenced by information bias, due to social desirability (i.e., it is easier to admit lack of physical activity than alcohol abuse). Third, we considered in our analysis only lifestyle risk factors with convincing evidence for causing cancer, and for which exposure data and dose-response relationship of exposure and cancer were available. This methodological approach may have underestimated our PAF results. For instance, physical activity has been associated with endometrial cancer [57] and other sites of cancer [58], but the dose response relationship is still not well established. Tobacco smoking is causally associated with mucinous ovarian cancer; however, there is lack of adequate occurrence data for this type of cancer in Chile. Other modifiable risk factors, such as infectious agents (i.e., helicobacter pylori, hepatitis B virus, hepatitis C virus, and human papilloma virus) [59] and occupational exposures (i.e., asbestos, nickel and wood dust) [60], increase the risk of several cancer sites and therefore should also be considered for cancer prevention strategies in Chile.

## Conclusions

In Chile, around three in ten of all cancer cases and 36% of all cancer deaths in 2018 were attributable to lifestyle risk factors. Smoking and high BMI were the leading causes of preventable cancers, followed by alcohol consumption, lack physical activity, low consumption of fruits and vegetables and passive smoking. Cancer prevention strategies should consider evidence-based interventions and public policies to reduce exposure and encourage the adoption of a healthier lifestyle.

## Data Availability

The datasets generated and/or analysed during the current study are available in the database repository of the Epidemiology Department of the Chilean Ministry of Health: http://epi.minsal.cl/bases-de-datos/

## Abbreviations

BMI: Body mass index
ENS: Encuesta Nacional de Salud;
IARC: International Agency for Research on Cancer
MET: Metabolic equivalent tasks
NCP: National Cancer Prevention
NHSC: National Health Survey of Chile
PAF: Population attributable fraction
RR: Relative risks
WCRF: World Cancer Research Fund
WHO: World Health Organization
